# Brief Negative Affect Focused Functional Imagery Training Abolishes Stress-Induced Alcohol Choice in Hazardous Student Drinkers

**DOI:** 10.1155/2021/5801781

**Published:** 2021-09-17

**Authors:** Alexandra Elissavet Bakou, Ruichong Shuai, Lee Hogarth

**Affiliations:** School of Psychology, University of Exeter, Exeter, UK

## Abstract

**Introduction:**

Imagery-based stress management therapies are effective at reducing alcohol use. To explore the therapeutic mechanism, the current study tested whether brief functional imagery training linked to personal negative affect drinking triggers would attenuate sensitivity to noise stress-induced alcohol seeking behaviour in a laboratory model.

**Methods:**

Participants were UK-based hazardous student drinkers (*N* = 61, 80.3% women, aged 18–25) who reported drinking to cope with negative affect. Participants in the active intervention group (*n* = 31) were briefly trained to respond to personal negative drinking triggers by retrieving an adaptive strategy to mitigate negative affect, whereas participants in the control group (*n* = 30) received risk information about binge drinking at university. The relative value of alcohol was then measured by preference to view alcohol versus food pictures in two-alternative choice trials, before (baseline) and during noise stress induction.

**Results:**

There was a significant two-way interaction (*p* < .04) where the control group increased their alcohol picture choice from baseline to the noise stress test (*p* < .001), whereas the active intervention group did not (*p*=.33), and the control group chose alcohol more frequently than the active group in the stress test (*p*=.03), but not at baseline (*p*=.16).

**Conclusions:**

These findings indicate that imagery-based mood management can protect against the increase in the relative value of alcohol motivated by acute stress in hazardous negative affect drinkers, suggesting this mechanism could underpin the therapeutic effect of mood management on drinking outcomes.

## 1. Introduction

Hazardous drinking among UK university students is a serious public health concern [1, 2]. Motivational models of alcohol dependence suggest that negative affective states, such as feelings of stress, depression, and anxiety, powerfully motivate alcohol use in order to mitigate these negative states not only in clinical samples but also among student drinkers who drink to cope [3–7]. Causal evidence for this claim comes from the finding that experimental negative affect induction reliably increases alcohol demand, i.e., willingness to spend money on alcohol [8], and increases preferential choice of alcohol over alternative reinforcers, indicating that negative affect raises the relative value of alcohol over other rewards in university students [9, 10]. Furthermore, such effects are augmented in individuals who self-report drinking to cope (DTC) with negative affect suggesting that individual differences in sensitivity to negative affect priming of alcohol choice may be a core component of dependence [11]. Based on evidence such as this, clinical mood management therapies seek to attenuate the impact of negative affect on motivation to drink [12–17]. Although such therapies improve drinking outcomes, it is not known whether this therapeutic effect is mediated by reduced sensitivity to negative affect priming of alcohol choice behaviour.

One therapeutic mood management strategy has been to provide personalised feedback about an individual's drinking to cope (DTC) to raise awareness of their negative drinking triggers. For example, Blevins and Stephens [18] provided undergraduates with feedback on their DTC versus standard alcohol risk feedback. At the 2-month follow-up, the DTC feedback group reported a reduction in drinking to cope compared to controls, and both groups showed a comparable reduction in drinking frequency. However, the reduction in DTC mediated the reduction in drinking frequency in the DTC feedback group but not the control group, suggesting the intervention may have modified drinking frequency by reducing DTC cognitions [18]. Similarly, Wurdak et al. [19] developed a brief drinking motives tailored intervention that was delivered to adolescents admitted to hospital for acute alcohol intoxication. Adolescents who endorsed DTC were trained on a relaxation technique providing an adaptive response to stress. Compared to participants who received standard medical advice, female participants who received this motives-tailored intervention reported lower drinking frequency and less binge drinking at one-month follow-up. Finally, Conrod et al. [20] screened and identified younger school adolescents (largely prior to alcohol use onset) who reported anxiety sensitivity or hopelessness and trained them on goal setting exercises to promote adaptive responses to these emotions. Compared to the control group, the personality tailored intervention was effective in slowing the growth of drinking outcomes at 24 months [20–22]. Thus, increasing awareness of negative drinking triggers and linking them to adaptive strategies in at risk DTC drinkers can improve drinking outcomes, but the exact therapeutic mechanism remains unknown.

Episodic Positive Future Thinking (EPFT), Functional Imagery Training (FIT), and “Best Possible Self” interventions all train clients to retrieve a mental image of possible positive future activities. These positive imagery-based interventions have been shown to decrease negative affect and raise positive affect [23–26]. These interventions also reduce delay discounting [27–31], food consumption [32–38], and tobacco and alcohol demand [27, 30, 39–41]. One interpretation of these effects is that imagining positive future activities reduces sensitivity to negative affect motivated consummatory behaviour, but this mechanism has not been tested directly.

The current study tested whether stress-induced alcohol seeking in a laboratory model would be attenuated by brief functional imagery training (FIT) in which hazardous DTC drinker were taught to respond to their personalised negative affect drinking triggers by imagining a positive future activity, compared to a control group that received risk information about binge drinking at university. To test this, following brief FIT versus control intervention, preferential choice between alcohol and food pictures was measured across a series of choice trials, to index the relative value of alcohol at baseline. This concurrent pictorial alcohol choice task has been previously validated as a measure of alcohol value insofar as preferential alcohol choice reliably correlates with alcohol dependence severity [11] and can be increased by stress and sadness induction [9, 42, 43]. Finally, all participants were stressed with a loud unpleasant noise, and concurrent choice of alcohol versus food pictures was measured again, to index the stress-induced increase in alcohol choice relative to baseline. The question addressed was whether the brief FIT intervention would attenuate the stress-induced increase in alcohol choice, supporting the claim that this mechanism could underpin the therapeutic effect of imagery-based mood management strategies on drinking outcomes.

## 2. Methods

### 2.1. Participants and Screening Questionnaires

Participants were 64 hazardous drinkers who endorsed drinking to cope, recruited from the University of Exeter student population. A brief online survey delivered via Qualtrics established whether participants met inclusion criteria: aged 18–25, scored ≥8 in the Alcohol Use Disorder Identification Test (AUDIT) indicating hazardous drinking, and endorsed 5 or more items on the Drinking to Cope Checklist (DTCC) indicating negative affect drinking [44]. The AUDIT contains 10 items assessing the frequency of alcohol use and alcohol-related problems experienced in the past 12 months. Total scores range from 0 to 40 split into categories: low-risk (0–7), hazardous (8–15), harmful (16–19), and possibly dependent (20–40) [45]. The Drinking to Cope Checklist (DTCC) lists 35 negative states which participants endorse as motivating their drinking with a yes/no response. In a prior validation sample of 488 Exeter students, 5 was the median number of items endorsed, so this criterion was chosen to select the top half of negative affect drinkers. Participants provided informed consent prior to the lab session, were fully debriefed, and received £5 as reimbursement for participation. The study was approved by the School of Psychology Research Ethics Committee.

### 2.2. Baseline Questionnaires

Eligible participants were scheduled for a face-to-face experimental session where they completed the following baseline measures: the Daily Drinking Questionnaire-Revised (DDQR) adapted from [46] in which participants reported each alcoholic drink type and volume they consumed for each of the last 14 days, which was converted into standard alcohol units and summed; the PROMIS Alcohol Use Short Form [47], which includes 7 items referring to drinking in the past two weeks (e.g., “I drank more than I planned”); the modified five factor Drinking Motives Questionnaire Revised (DMQR), which measures how frequently drinking is motived by each listed reason, on a 1–10 scale ranging from “never” to “almost always” [48]; this questionnaire has five subscales: drinking to cope with anxiety and depression, conformity, enhancement, and socialising. The depression and anxiety subscales were highly correlated, *r* (59) = .78, *p* < .01, and so were collapsed into a single coping subscale; the Generalised Anxiety Disorder (GAD) [49] scale assessing GAD symptoms in the past two weeks (e.g., “feeling nervous, anxious or on edge”). The score on each item ranges from 0 (“not at all”) to 3 (“nearly every day”) and total scores range from 0 to 21, with a score of 5, 10, and 15 as the cut-off points for mild, moderate, and severe anxiety, respectively; the Patient Health Questionnaire depression scale (PHQ) [50] which contains 8 items assessing depressive symptoms in the past two weeks (e.g., “little interest or pleasure in doing things”). The score on each item ranges from 0 (“not at all”) to 3 (“nearly every day”) and total scores range from 0 to 24, with a score of 5, 10, 15, and 20 as the cut-off points for mild, moderate, moderately severe, and severe depression, respectively.

### 2.3. Active versus Control Intervention

Interventions were delivered by a brief video of a PowerPoint presentation containing text and images, and text read out over headphones by a female voice (the full video is here: https://youtube/xbhTxFCcRjY). The active video contained 13 pages communicating two points summarised by the opening statement: “your questionnaire responses indicated that you are at increased risk of alcohol dependence in the future. This is because you drink to cope with negative emotions, such as depression, stress, and boredom. This video will teach you a ‘reactive imagery' technique, to deal with negative emotions, which will help reduce your risk of alcohol dependence in the future.” Participants were then provided with a personalised list of the negative affective states that they had endorsed as motivating their drinking in the DTCC and that they should be aware of these triggers (“look at your questionnaire responses on the right. You said that you drink to cope with all the negative emotions listed. Please take a moment to look through the negative emotions that trigger your drinking”). Participants were then trained on the imagery technique summarises by the statement: “the reactive imagery technique is very simple: when you experience negative emotions (such as those you would drink to cope with), react by vividly imagining your best self—you on a good day—and ask ‘what would your best self do now?'.”

The control video was 4 minutes (8 pages) of text presented and read out summarising the binge drinking risk information from the US National Institute on Alcohol Abuse and Alcoholism (NIAAA) College Drinking Factsheet (https://www.niaaa.nih.gov/publications/brochures-and-fact-sheets/college-drinking; the full video is here: https://youtube/08ra9hBqcf4).

### 2.4. Alcohol Choice at Baseline

After the intervention manipulation, participants completed a pictorial choice task in which they freely chose to enlarge thumbnail pictures of either alcohol or food by pressing a left or right arrow key across a series of choice trials [9, 10]. Instructions were as follows: “In this task, you can view alcohol and food pictures by pressing the left or right arrow key.” In each of the 24 baseline trials, an alcohol and a food thumbnail stimulus were each sampled randomly from a set of 28 alcohol and 28 food images and presented randomly in the left or right screen position. Each thumbnail pair remained until a left or right arrow key press enlarged the selected image which remained on the screen for 2 seconds followed by a 1-second intertrial interval. Following baseline alcohol choice, subjective mood was measured to provide a validation check for the stress induction protocol. Participants were asked to what extent they currently felt ‘happy' and ‘annoyed', in random order, on a 5-point scale ranging from 1 (“not at all”) to 5 (“extremely”). All participants then completed the choice task during noise stress, i.e., another 24 pictorial choice trials while a loud (75 dB) industrial noise was played continuously through headphones to induce mild stress and increase alcohol choice [10]. Subjective mood was measured again afterwards to quantify change in subjective mood from baseline.

### 2.5. Analytical Plan

Percent alcohol versus food image choice was calculated from baseline and test trials and entered into mixed 2 × 2 ANOVA with the between-subjects variable intervention group (active, control) and the within subjects variable time point (baseline, stress test). Subjective happiness and annoyance were entered into separate mixed 2 × 2 ANOVAs with the same variables. A significant interaction would indicate that the intervention modified the stress-induced change in alcohol choice or mood.

## 3. Results

### 3.1. Participants

One hundred and eighty-three participants responded to the recruitment ad and one hundred and forty-eight participants completed the online screening questionnaires. Seventy-four participants met the eligibility criteria and were all invited to come to the lab for the experimental session. Five participants failed to show up for the experiment and we experienced technical difficulties with data collection software with five of the participants at the beginning of the study which resulted in the experimental session being terminated. Three participants were excluded due to them having an outlying change in percent alcohol picture choices from baseline to test (greater than three times of the interquartile range, with two outliers above and one outlier below these boundaries), leaving 61 participants for analysis. Participant characteristics are shown in Table 1—on all of which the active and control group were matched.

### 3.2. Percent Alcohol Picture Choice

**Figure 1:**
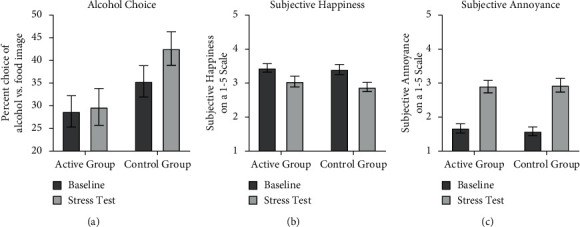
(a) Percent choice of alcohol versus food images during the baseline and stress phases in the active and control group. The active intervention abolished the stress-induced increase in alcohol choice found in the control group. Subjective happiness (b) and annoyance, (c) immediately following the baseline and stress choice phases in the active and control group. Stress induction worsened subjective mood but the intervention manipulation did not modify these effects. Error bars in this figure represent standard errors.

Figure 1(a) shows the percent alcohol picture choice measured at baseline and in the stress test for the active and the control group. There was a significant interaction, *F* (1, 59) = 4.59, *p*=.036, *η*^2^=.72, driven by a significant increase in alcohol choice from baseline to stress test in the control group, *F* (1, 29) = 21.17, *p* < .001, *η*^2^=.42, but not in the active group, *F* (1, 30) = 0.97, *p*=.33, *η*^2^=.03. Furthermore, the two groups did not significantly differ at baseline, *F* (1, 60) = 1.98, *p*=.16, *η*^2^=.03 but did differ significantly in the stress test, *F* (1, 60) = 4.51, *p*=.038, *η*^2^=.07.

### 3.3. Subjective Mood

For subjective happiness, shown in Figure 1(b), there was a significant main effect of time point, *F* (1, 59) = 33.3, *p* < .001, *η*^2^=.36, and no significant main effect of group, *F* (1, 59) = .75, *p*=.38, *η*^2^=.01 or interaction between group and time point, *F* (1, 59) = .84, *p*=.36, *η*^2^=.01. For annoyance, shown in Figure 1(c), there was again a significant main effect of time point, *F* (1, 59) = 75.2, *p* < .001, *η*^2^=.56, and no significant main effect of group, *F* (1, 59) = .04, *p*=.82, *η*^2^=.00, or interaction between group and time point, *F* (1, 59) = .22, *p*=.63, *η*^2^=.00. These findings indicate that stress induction decreased happiness and increased annoyance comparably in both groups, indicating that the active intervention did not protect against stress-induced worsening of subjective mood.

## 4. Discussion

The study found that a brief active intervention comprising awareness of personal negative affect drinking triggers and training to retrieve a mental image of an adaptive strategy in response to those negative affect triggers selectively abolished the noise stress-induced increase in alcohol choice behaviour in hazardous student drinkers who drink to cope. There was no difference between the active and control group in alcohol choice at baseline, suggesting the intervention did not change the value of alcohol directly, but rather selectively abolished the stress-induced increase in alcohol choice measured in the stress test, suggesting specificity of the intervention effect. Furthermore, both groups were equally sensitive to the effects of stress induction on worsening of subjective mood (reduced happiness and increased annoyance), suggesting that the therapeutic effect of the active intervention did not extend to stress-induced changes in subjective mood.

As noted in the introduction, various DTC and imagery focused interventions have been shown to reduce drinking outcomes [18, 19, 21], eating behaviour, and demand for tobacco and alcohol [27, 30–32, 39, 40, 51]. One proposed therapeutic mechanism is that these interventions attenuate sensitivity to negative affect induced changes in alcohol motivation. The novel contribution of the current study was to demonstrate that the brief intervention which incorporated elements of these therapies did selectively attenuate stress-induced alcohol choice, suggesting these therapies could impact on drinking outcomes via this mechanism. To fully corroborate this claim about the therapeutic mechanism, mediation studies are needed within randomised controlled trials, to test whether therapeutic effects on drinking outcomes are mediated by a reduction in sensitivity to stress-induced alcohol choice [52].

There were a number of limitations in the study. First, the sample size was small; future studies with a larger sample size are needed to further explore the effects of this intervention. This is important because although there was no significant difference between groups in alcohol choice in the baseline test, there was a numeric difference. The control group's choice percentage was 35.55%, which was greater than the intervention group at 28.76%. Thus, it remains possible that the two intervention manipulations could have differential effects on alcohol choice in the absence of stress, but this effect might only emerge with a larger sample size and thus greater power. Secondly, because the active group included two active elements (feedback + imagery) compared to the control group, we cannot isolate whether one or other element, or their interaction, was crucial for the effect on resilience to stress-induced alcohol choice. Future studies are needed in which the two elements are isolated and combined in separate groups, to test their independent and interactive effects.

Third, the study sample was composed of adult student drinkers. Studies suggest differential pathways in the experience of negative affect and drinking to cope between student and non-student drinkers and, as such, our findings may not extend to non-students [53, 54]. Fourth, our sample was predominantly female and so it remains unknown whether similar therapeutic effects would be seen in males [19].

Perhaps the greatest concern is the possibility that the effects were driven by demand characteristics, that is, by participants' beliefs about what the experimenter wants rather than a true therapeutic effect. The active intervention sought to instill an explicit instruction in active participants to think of a positive future activity when experiencing negative affect, and if they did this in the stress test in response to the noise, it would have selectively attenuated the stress-induced alcohol choice as found. The demand characteristics account, by contrast, suggests that instead of thinking about a positive future in the stress test participants inferred that the experimenter expected lower alcohol choice in that phase, and so did not increase their responding relative to baseline (unlike the control group). It is curious that responding remained stable from baseline to the stress test in the active group, as the demand account might anticipate a decrease if one inferred that was what the experimenter wanted. The strongest evidence of a true therapeutic effect of the brief intervention would be to demonstrate a change in drinking outcomes in a clinical trial. In fact, the same brief intervention was employed in a small scale, online, randomised controlled trial (RCT) with hazardous, student, negative affect drinkers, who practiced the reactive imagery over 2 weeks [55]. It was found that the active intervention group increased self-efficacy of control over negative affect drinking and alcohol consumption and decreased social drinking motives from baseline to two-week follow-up, relative to a control group (akin to the present). However, there were no group differences in drinking frequency. The current study and the small-scale RCT provide converging evidence that active intervention has some true therapeutic efficacy, in attenuating stress-induced alcohol seeking and increasing confidence in control over negative affect drinking. The next stage of the research should be to test whether the brief intervention can attenuate actual drinking behavioural outcomes, and the extent to which this effect is mediated by a reduction in stress-induced alcohol choice in the current experimental model, quantifying the role of this therapeutic mechanism in changing drinking behaviour [56].

In conclusion, this study found that the deployment of a brief negative affect focused positive imagery intervention abolished stress-induced increases in alcohol choice in a sample of hazardous student negative affect drinkers. This finding offers a proof of concept for this brief intervention and supports the claim that DTC/imagery-based interventions might operate by building resilience to negative affective drinking triggers.

## Figures and Tables

**Table 1:** Mean (SD, range) of questionnaire data reported by the active intervention and control groups. AUDIT = Alcohol Use Disorder Identification Test. PROMIS  =  Patient-Reported Outcomes Measurement Information System Alcohol Use Short Form. DMQR = modified Drinking Motives Questionnaire Revised. GAD = Generalised Anxiety Disorder test. PHQ  =  Patient Health Questionnaire depression scale. *p* = significance level of the group contrast.

	Group		*p*
	Active (*N* = 31)	Control (*N* = 30)	
Age	20.1 (1.84, 18–25)	19.6 (1.97, 18–25)	.78
Gender (% female students)	83.8%	76.6%	.16
AUDIT	21.6 (4.59, 16–35)	21.2 (3.40, 16–29)	.21
Alcohol units (past 14 days)	36.7 (27.30, 6.30–149.7)	38.4 (22, 11.50-89.90)	.80
PROMIS	16.8(4.09, 10–25)	15.8 (4.16, 10–24)	.82
DMQR coping	5.3 (1.86, 1.25–8.85)	5.5 (2.07, 1.97–10.19)	.36
DMQR conformity	3.4 (1,97, 1–7.4)	4.5 (2.56,1–9.80)	.14
DMQR social	8.5 (1.59, 2.8–10.6)	8.2 (1.33, 4–10.4)	.52
DMQR enhancement	7.1 (1.99, 2.4–10.4)	7.7 (1.84, 3–11)	.54
GAD-7	5.8 (3.90, 0–15)	5.8 (4.09, 0–18)	.76
PHQ-8	7.2 (4.71, 1–21)	6.9 (3.78,0–18)	.28

## Data Availability

Data are available on request.
